# Impact of epidural analgesia on the systemic biomarker response after hepatic resection

**DOI:** 10.18632/oncotarget.26549

**Published:** 2019-01-15

**Authors:** Diego Vicente, Miguel Patino, Rebecca Marcus, Heather Lillmoe, Preparim Limani, Timothy Newhook, Andy Lee, Ching-Wei Tzeng, Yun Segraves-Chun, David Tweardy, Vijaya Gottumukkala, Jean-Nicolas Vauthey, Thomas Aloia, Juan P. Cata

**Affiliations:** ^1^ Department of Surgical Oncology, The University of Texas MD Anderson Cancer Center, Houston, TX, USA; ^2^ Anesthesiology and Surgical Oncology Research Group, Houston, TX, USA; ^3^ Department of Molecular and Cellular Oncology, The University of Texas MD Anderson Cancer Center, Houston, TX, USA; ^4^ Department of Anesthesiology and Perioperative Medicine, The University of Texas MD Anderson Cancer Center, Houston, TX, USA

**Keywords:** analgesia, epidural, cytokines

## Abstract

**Background:**

Perioperative inflammation is associated with poor oncologic outcomes. Regional analgesia has been shown mitigate some of these inflammatory changes and be associated with better oncologic outcomes in patients with hepatic malignancies. The mechanism for this effect, however, remains unclear. The authors sought to compare systemic biomarker concentrations in a comprehensive and oncologically relevant panel in the perioperative setting between patients undergoing thoracic epidural analgesia (TEA) and intra-venous patient- controlled analgesia (IV-PCA) for resection of hepatic metastatic disease.

**Results:**

Clinicopathologic variables and baseline biomarkers were similar between TEA (*n* = 46) and IV-PCA (*n* = 16) groups. Of the biomarkers which were significantly changed from baseline, there was a lower fold change from baseline in the TEA patients compared to IV-PCA including IL-6 (13.5vs19.1), MCP-1 (1.9vs3.0), IL-8 (2.4vs3.0), and Pentraxin-3 (10.8vs15.6). Overall decreased systemic concentrations of TGFb signaling were noted in TEA patients on POD1 TGFb3 (243.2 vs. 86.0, *p* = 0.005), POD3 TGFb1 (6558.0 vs. 2063.3, *p* = 0.004), POD3 TGFb2 (468.3 vs. 368.9, *p* = 0.036), POD3 TGFb3 (132.2 vs. 77.8, *p* = 0.028), and POD5 TGFb3 (306.5 vs. 92.2, *p* = 0.032). POD1 IL-12p70 concentrations were significantly higher in TEA patients (8.3 vs. 1.6, *p* = 0.024).

**Conclusion:**

Epidural analgesia damped the postoperative inflammatory response and systemic immunosuppressive signaling, as well as promoted Th1 systemic signaling early in the post-operative period after hepatic resection for metastatic disease. These differences elaborate on known mechanisms for improved oncologic outcomes with regional anesthesia, and may be considered for biomarker monitoring of effective regional anesthesia in oncologic surgery.

**Materials and Methods:**

Patient data, including clinicopathologic variables were collected for this study from the database of a randomized controlled trial comparing perioperative outcomes in patients undergoing hepatic resection with TEA vs. IV-PCA. Patients undergoing resection for metastatic disease were selected for this study. Plasma concentrations (pg/mL) of well-studied biomarkers (IL-1b/2/4/5/6/7/8/10/12p70/13/17, MCP-1 IFNγ, TNFα, MIP-1b, GM-CSF, G-CSF, VEGF, Resistin, TGFb1, TGFb2, and TGFb3), as well as novel perioperative markers (CXCL12, CXCL10, Omentin-1, sLeptin R, Vaspin, Pentraxin-3, Galactin-3, FGF-23, PON-1, FGF-21) were measured preoperatively, and on postoperative day (POD)1, POD3, and POD5 using multiplex bead assays. Clinicopathologic variables and perioperative variations in these biomarkers were compared between TEA vs IV-PCA groups.

## INTRODUCTION

Hepatic resection for metastatic disease has improved oncologic outcomes in various solid organ malignancies [1]. Despite advances in technique [2] and appropriate oncologic resections [3] for these patients, perioperative inflammatory stimuli [4] and management strategies such as volatile anesthetic agents [5] and opioid analgesia [6] have been implicated in poor oncologic outcomes [7–9]. These factors are associated with suppression of cell mediated immunity, shift from Th1 to Th2 signaling, augmented tumor growth, and pro-metastatic systemic signaling [10]. In some cancers, use of regional analgesia in surgical oncology procedures has been associated with increased disease-free survival (DFS) and overall survival (OS) compared to traditional post-operative opioid-based analgesia [7, 9, 11, 12]. The mechanism for these improved oncologic outcomes, however, remains unclear.

Current literature evaluating the impact of epidural analgesia on lymphocyte function [13–15], Th1 vs. Th2 balance [16], and systemic markers of inflammation [17–20] has yielded mixed results. Furthermore, oncological relevant biomarkers to measure the impact of regional anesthesia on the response to surgery have not been established. Herein, we evaluated the longitudinal postoperative impact of thoracic epidural analgesia (TEA) on a comprehensive systemic biomarker panel from patients undergoing elective hepatic resection for metastatic disease as part of a randomized controlled trial comparing TEA and intravenous patient controlled analgesia (IV-PCA). In addition to established perioperative biomarkers, a novel set of oncological relevant systemic markers involved in Th1 immune response (CXCL10) [21]; tumor associated macrophages (CXCL12) [22]; tumor suppression (Omentin-1) [23]; pro-metastatic signaling (Galectin-3) [24]; and tumor associated inflammation (Pentraxin-3) [25] were evaluated. Our hypothesis was that TEA would dampen the inflammatory response, preserve Th1 biomarkers, and decrease pro-metastatic signaling in the postoperative period in comparison to IV-PCA.

## RESULTS

Table 1 demonstrates that clinicopathologic variables were overall similar between patients in the TEA (*n* = 45) and IV-PCA (*n* = 16) groups. Metastatic pathology was also similar between groups and consisted predominantly of colorectal liver metastasis (75 vs. 93%, *p* = 0.070). Additional metastatic disease pathology included neuroendocrine tumor (*n* = 2), sarcoma (*n* = 1), adrenocortical carcinoma (*n* = 1), ovarian carcinoma (*n* = 1), medullar thyroid cancer (*n* = 1), and breast cancer (*n* = 1) distributed between the two groups. During the hospitalization, the IV-PCA group had an increased opioid consumption compared to the TEA group (386.3 [233.8–588.5] vs. 154.3 [99.4–264.2] mg, *p* = 0.012).

**Table 1:** Clinicopathologic, operative, and recovery variables of patients in all patients, as well as PCA and TEA groups

	Epidural vs. PCA				
	PCA *n* = 16		TEA *n* = 45		
	*n* or median	% or IQR	*n* or median	% or IQR	*p*
**Age**	61	52–64	55	48–61	0.166
**Male**	8	50%	27	60%	0.563
**ASA = 3**	13	81%	41	91%	0.365
**BMI**	24	23-30	27	24-30	0.425
**Received Pre-operative Chemotherapy**	11	69%	39	89%	0.112
**Portal Vein Embolization**	0	0%	5	11%	0.313
**Major Hepatectomy**	9	56%	30	66%	0.568
**Midline Incision**	9	56%	31	68%	0.376
**Operative Time (min)**	259	220–316	260	206–306	0.516
**Estimated Blood Loss (mL)**	200	150–375	200	100–300	0.343
**Perioperative Transfusion**	2	12%	4	9%	0.648
**Postoperative Complication**	5	31%	16	36%	0.999
**Length of Stay (days)**	5	5–6	6	5–7	0.663
**PCA or Epidural Duration (days)**	3	3–4	4	3–4	0.857
**Colorectal Liver Metastasis**	12	75%	42	93%	0.070
**Opioid Consumption (mg)**	386.3	233.8–588.5	154.3	99.4–264.2	0.012
**Inadequate Pain Control**	8	50%	17	38%	0.555

Patients in the TEA group had lower opioids requirement during their hospitalization, but the groups were otherwise similar.

Preoperative concentrations of biomarkers are shown in Table 2. IL-6 (35.2 vs. 10.1, *p* = 0.048) was noted to be higher in the IV-PCA group, while the remainder of the biomarkers were similar between the two groups prior to surgery. Of note, poor signal quality outside of the standard’s concentration range was noted in IL-1b, IL-2, IL-4, IL-5, IL-7, IL-10, IL-13, IL-17, VEGF, GM-CSF, and G-CSF before surgery and throughout the experiment in most patients.

**Table 2:** Preoperative plasma biomarker levels in all patients

	Analgesic Technique		*P* value
	PCA (*n* = 16)	Epidural (*n =* 45)	
	Median (IQR)	Median (IQR)	
**IL6**	35.2 (12.5, 52.1)	10.1 (6.4, 20.7)	0.048
**IL8**	93.9 (35.7, 158.5)	82.8 (50.9, 96.0)	0.432
**IL12p70**	4.8 (3.4, 8.5)	7.7, (4.8, 13.1)	0.899
**CXCL10**	108.9 (58.4–355.1)	94.9 (62.5, 178.4)	0.679
**CXCL12**	1432.8 (529.9, 5006.3)	958.5 (666.2, 1370.3)	0.163
**INFg**	26.7 (11.0, 71.3)	6.9 (2.8, 14.3)	0.137
**TNFa**	116.5 (72.9, 201.3)	128.6 (96.7, 199.6)	0.433
**TGFb1**	4078.9 (2961.3, 8302.6)	2056.8 (478.1, 4294.1)	0.087
**TGFb2**	417.6 (272.7, 512.0)	271.9 (102.9, 409.9)	0.181
**TGFb3**	173.9 (41.7, 908.9)	57.5 (48.8, 151.4)	0.164
**MIP1b**	80.4 (49.3, 173.1)	98.3 (64.6, 137.3)	0.794
**MCP1**	280.1 (174.6, 396.4)	254.6 (182.7, 418.7)	0.681
**Resistin**	8650.2 (6040.7, 10705.6)	5705.9 (4196.3, 8461.1)	0.160
**Omentin1**	352746.9 (129012.2, 484118.7)	512229.7 (94982.9, 829895.6)	0.351
**Pentraxin3**	1191.0 (811.4, 2273.2)	1531.8 (683.4, 3579.1)	0.456
**Galactin3**	8572.8 (6224.2, 16169.2)	13568.6 (11411.1, 16902.5)	0.176
**sLeptinR**	10208.3 (1702.8, 15521.7)	10687.7 (2835.3, 13680.6)	0.341
**Vaspin**	87.0 (46.7, 300.3)	111.9 (62.1, 288.5)	0.856
**FGF21**	138.9 (41.9, 595.9)	131.2 (65.4, 201.3)	0.221
**FGF23**	88.3 (42.2, 195.2)	91.7 (59.4, 149.6)	0.276
**PON1**	2710100.0 (1831150.0, 7488825.0)	6833800.0 (3983625.0, 9489499.0)	0.069

IL-6 was elevated in the PCA group. Of note biomarkers IL1b, IL2, IL4, IL5, IL7, IL10, IL13, IL17, VEGF, GMCSF, GCSF demonstrated poor signal quality at baseline and throughout the experiment.

**Figure 1:**
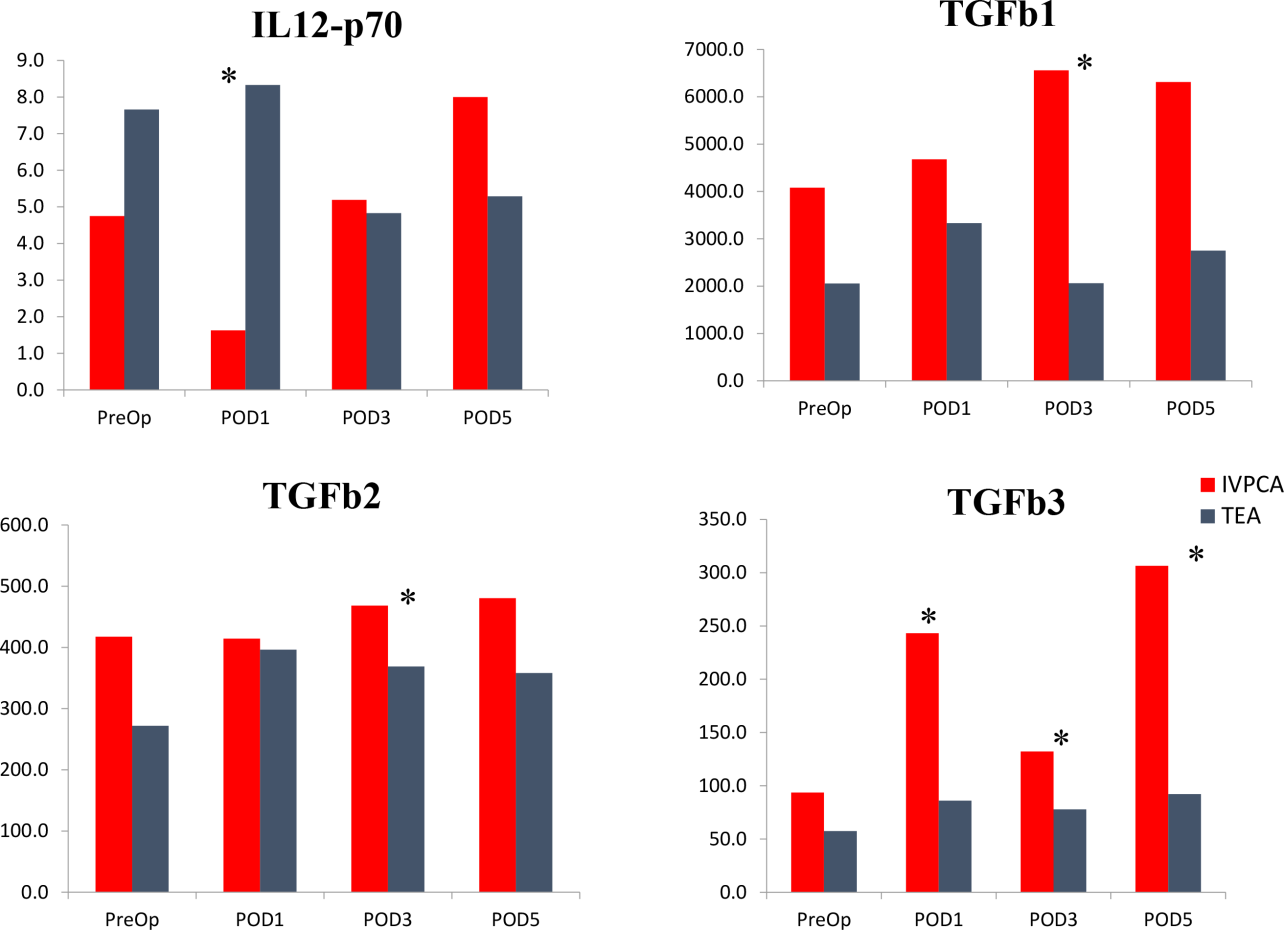
Concentrations (pg/mL) of the biomarkers which were significantly different between TEA and PCA groups in the postoperative time period There were no significant differences between postoperative levels of remaining biomarkers. PreOp - preoperative. POD – postoperative day. ^*^indicates significant differences between TEA and IVPCA patients (*p* < 0.05).

Considering the concentrations of biomarkers throughout the study, statistically significant differences between groups were noted in TGFb1/2/3 and IL-12p70 as demonstrated in Figure 1. Compared to the IV-PCA group, the TEA group had significantly lower TGFb3 levels on POD1 (243.2 vs. 86.0 pg/mL, *p* = 0.005), POD3 (132.2 vs. 77.8 pg/mL, *p* = 0.028), POD5 (306.5 vs. 92.2 pg/mL, *p* = 0.032). On POD3, TGFb1 (6558.0 vs. 2063.3 pg/mL, *p* = 0.004) and TGFb2 (468.3 vs. 368.9 pg/mL, *p* = 0.036) were also significantly lower in TEA group. The POD1 IL-12p70 was significantly higher in TEA patients (8.3 vs. 1.6 pg/mL, *p* = 0.024).

In order to evaluate significant variation from preoperative values and fold change in all detectable biomarkers, we compared the concentrations of the biomarkers on POD1, POD3, and POD5 to preoperative values in all patients as seen in Table 3. Of note MIP1b, INFγ, TNFα, IL-17, IL-12p70, CXCL12, Omentin1, sLeptinR, Vaspin, Galactin3, FGF23, PON1, FGF21, TGFb1, TGFb2, and TGFb3 did not vary significantly from preoperative concentrations. The peak fold changes for most biomarkers occurred on POD1, with the largest median fold changes noted in IL-6 (14 folds) and Pentraxin-3 (12 folds). CXCL10 values decreased below baseline (0.48 folds) on POD1. Most biomarker values began to trend back to baseline by POD3.

**Table 3:** Levels of fold change in detectable biomarkers in all patients

	PreOp	POD1			POD3			POD5		
	Median (IQR)	Median (IQR)	Fold	*p*	Median	Fold	*p*	Median	Fold	*p*
**IL6**	13.0 (8.0, 34.7)	73.6 (30.4, 202.9)	14.852	0.052	32.2 (16.7, 49.9)	5.857	0.041	16.6 (12.5, 31.3)	2.582	0.320
**IL8**	82.75 (48.6, 118.5)	151.0 (93.9, 280.9)	2.554	0.000	112.4 (83.3, 158.6)	1.726	0.046	115.4 (88.9, 156.4)	1.638	0.052
**IL12p70**	7.2 (3.7, 12.5)	7.7 (3.2, 10.2)	0.773	0.137	4.8 (2.8, 10.0)	0.678	0.918	6.5 (1.8, 10.1)	1.063	0.135
**CXCL10**	101.1 (61.5, 232.2)	51.5 (36.2, 116.1)	0.487	0.040	79.2 (51.9, 136.4)	0.946	0.209	97.7 (41.7, 203.5)	0.857	0.492
**CXCL12**	965.15 (614.3, 1539.0)	894.8 (534.4, 1255.4)	0.730	0.362	1207.4 (628.6, 1925.9)	1.022	0.854	1068.9 (614.1, 1824.6)	1.039	0.965
**INFg**	11.0 (3.4, 23.6)	11.1 (6.3, 22.6)	1.734	0.549	10.7 (5.9, 22.2)	1.492	0.640	12.7 (4.7, 41.8)	2.523	0.259
**TNFa**	126.0 (92.9, 202.6)	126.0 (81.9, 198.6)	0.974	0.459	135.1 (101.9, 227.5)	1.104	0.989	156.4 (95.5, 202.1)	1.090	0.805
**TGFb1**	3003.15 (1189.3, 5068.0)	3454.7 (2009.3, 5282.4)	2.788	0.376	2703.7 (1198.7, 6157.2)	2.224	0.926	2966.9 (1650.8, 4558.1)	1.299	0.639
**TGFb2**	306.2 (203.8, 442.1)	396.3 (206.1, 579.7)	1.485	0.545	402.9 (242.5, 543.7)	1.296	0.544	368.6 (238.1, 569.8)	1.114	0.503
**TGFb3**	63.3 (46.4, 210.5)	97.0 (77.4, 203.7)	1.010	0.556	94.9 (62.3, 186.1)	0.932	0.338	138.2 (65.0, 306.5)	0.904	0.643
**MIP1b**	95.7 (59.0, 143.9)	67.0 (48.9, 142.3)	0.962	0.645	95.8 (53.6, 141.9)	1.020	0.960	81.7 (55.0, 137.1)	1.083	0.558
**MCP1**	273.1 (182.7, 412.9)	451.9 (240.2, 782.5)	2.213	0.001	432.9 (288.7, 630.5)	2.017	0.015	341.1 (241.7, 564.3)	1.600	0.086
**Resistin**	6224.6 (4342.7, 9855.6)	13281.9 (7821.4, 20317.2)	2.420	0.000	7221.3 (5229.1, 10051.4)	1.357	0.201	7617.2 (4906.9, 11754.5)	1.360	0.050
**Omentin1**	435901.4 (115754.4, 771727.2)	504943.7 (133695.5, 932574.1)	1.303	0.321	318922.5 (45931.7, 604216.9)	0.695	0.703	129172.2 (14563.6, 346332.3)	0.613	0.534
**Pentraxin3**	1438.9 (677.6, 3385.8)	16795.8 (7074.2, 29856.8)	12.094	0.001	5185.2 (3203.2, 10193.4)	4.077	<0.001	4148.5 (1578.8, 8617.8)	2.497	0.124
**Galactin3**	12861.8 (8025.9, 16893.3)	13766.9 (10171.8, 17631.9)	1.148	0.683	12268.2 (8340.8, 15990.7)	1.037	0.297	11069.9 (6249.1, 16756.0)	1.114	0.498
**sLeptinR**	10687.72 (2290.0, 13971.2)	9570.9 (2053.1, 14689.6)	1.125	0.410	10503.6 (2535.6, 17330.9)	1.069	0.756	11196.5 (1928.2, 19685.4)	1.019	0.538
**Vaspin**	97.9 (56.9, 294.8)	39.7 (21.1, 92.0)	0.426	0.283	99.5 (56.7, 270.4)	0.986	0.455	121.7 (62.4, 231.5)	0.775	0.599
**FGF21**	131.2 (59.0, 335.9)	67.3 (27.5, 257.3)	1.267	0.867	117.3 (45.4, 347.7)	1.353	0.702	107.1 (48.5, 402.9)	1.313	0.890
**FGF23**	91.7 (49.6, 173.8)	76.8 (33.8, 181.8)	1.060	0.376	93.0 (51.423, 171.9)	0.956	0.520	122.6 (50.1, 361.7)	1.303	0.296
**PON1**	6567750.0 (2181700.0, 8901800.0)	5904950.0 (2512325.0, 8260475.0)	1.019	0.818	6009250.0 (3126650.0, 9958075.0)	1.132	0.582	3761700.0 (869938.9, 6759850.0)	0.844	0.479

IL-6, IL-8, MCP-1, Resistin, Pentraxin3, and CXCL10 were noted to be significantly different from baseline. Remainder of cytokines did not vary significantly from baseline. PreoOp: preoperative. POD: postoperative day.

**Figure 2:**
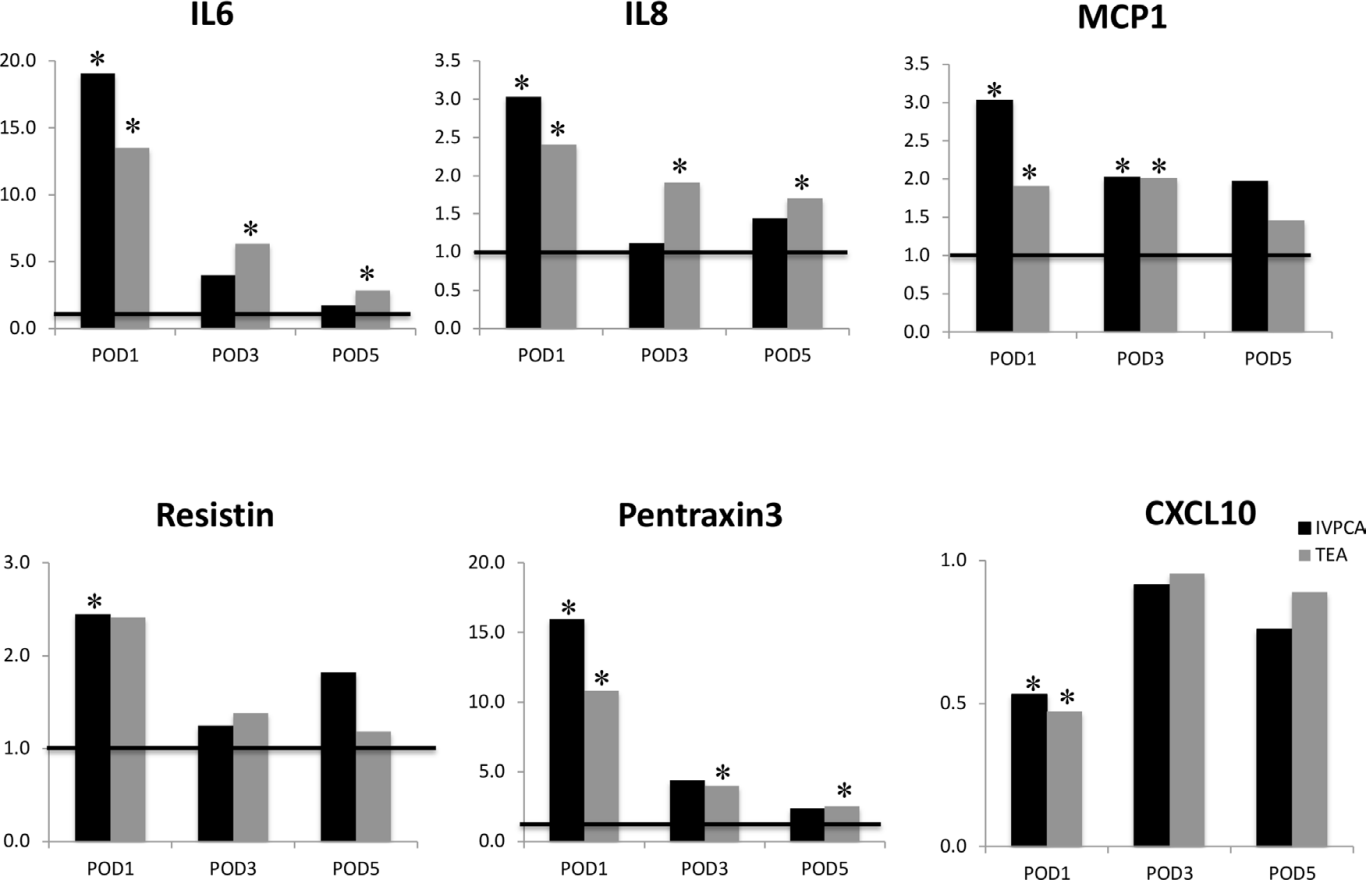
Mean fold change in biomarkers with significant variation from baseline in the IVPCA and TEA groups ^*^indicates significant difference from baseline in the TEA or the IVPCA group.

Of those biomarkers which varied significantly from baseline, Figure 2 illustrates that the TEA patients on POD1 had relatively lower mean fold changes from baseline compared to IV-PCA patients in IL-6 (13.5 vs. 19.1), MCP-1 (1.9 vs 3.0), IL-8 (2.4 vs 3.0), and Pentraxin-3 (10.8vs15.6) as well as similar increases in Resistin (2.4 vs. 2.4) and decrease in CXCL10 (0.47 vs. 0.53). Though most biomarkers trended back to baseline with similar fold changes in both groups on POD3 and POD5, the TEA group maintained IL-6, IL-8, MCP-1, and Pentraxin-3 levels that were significantly above baseline at these time points.

## DISCUSSION

In this comprehensive and longitudinal systemic biomarker analysis of patients undergoing elective hepatic resection for metastatic disease in a randomized controlled trial comparing TEA to IV-PCA, we demonstrated that patients with epidural analgesia had a favorable postoperative biomarker profile for oncologic surgery with reduction in TGFb concentrations, dampened response to surgery in fold changes of IL-6, IL-8, MCP-1 and Pentraxin-3, as well as increased values of IL-12p70. The remaining novel markers including CXCL12, Omentin-1, and Galectin-3 did not vary significantly from baseline and were not significantly different between the epidural and IV-PCA groups in the postoperative period.

The increase in Pentraxin3 is a novel finding in the elective perioperative setting, and to the authors knowledge, this is the first study to demonstrate an impact from regional analgesia in the postoperative fold change of this biomarker. Pentraxin3 is an important prognostic marker in sepsis [26] as well as colorectal cancer [25], and it is involved in the propagation of inflammation, tissue remodeling, angiogenesis, and tumor related inflammation [27]. Interestingly, Pentraxin3 demonstrated significant elevations relative to preoperative values in the postoperative period with large fold changes similar to IL6. IL6, IL8, and MCP1, followed the previously demonstrated postoperative kinetics with peak values on POD1 [28–31]. Beyond the immune response to injury, these are pluripotent biomarkers and also have significant oncologic impact as each has been associated with tumor burden in clinical studies [25, 32, 33, 65] as well as mechanisms involved in tumor progression in preclinical experiments [34–36].

While some studies have demonstrated that epidural analgesia is associated with lower postoperative levels of these biomarkers in various surgical procedures [18–20, 37–42], our data and supporting literature suggests that the overall concentrations of these proinflammatory markers were not different between TEA and IV-PCA patients [17, 28, 43]. These mixed results are likely associated with the large variations in inflammatory marker measurements prior to surgery and potential individual patient response to any intervention or stimuli. Interestingly, when considering the variations from baseline and fold change in these biomarkers, epidural analgesia was associated with an initial dampened proinflammatory response in IL6, IL8, MCP1, and Pentraxin3 on POD1, but also persistent elevation of these biomarkers above baseline on POD3 and POD5. This finding is unlikely due to postoperative inflammatory events given the similar complication rate, but rather this is in keeping with clinical findings suggesting that epidural analgesia is associated with early benefits of patient experience and pain control, but it may not decrease hospital length of stay and in some cases may slow patient recovery [44].

In concert with the inflammatory reaction to surgical trauma, TGFb is among the immunosuppressive signals produced early in the postoperative period to regulate inflammation [45]. In the setting of resection of metastatic disease, this surge in TGFb likely has both pro- and anti-oncogenic properties in various tissues in that it can induce apoptosis in cancer cells in early metastatic niches, but can also suppress macrophage phagocytic function [46], promote epithelial mesenchymal transition [47], stimulate angiogenesis and stromal proliferation in more established metastatic disease [19, 47–50]. Within the TGFb super family, TGFb1, TGFb2, and TGFb3 play roles in these oncogenic processes and elevated systemic TGFb levels have been found to be correlate to poor prognosis factor in colorectal cancer [51–53]. Though TGFb levels overall did not vary significantly from baseline in this study, TGFb1 demonstrated a median fold change >2 in the postoperative period and the levels were significantly lower in the TEA group. These findings are consistent with a similar randomized control trial, in which patients with epidural analgesia undergoing elective colon cancer resections had significantly lower TGFb1 levels early in the postoperative period [19]. Interestingly, the postoperative TGFb2 and TGFb3 levels remained relatively close to preoperative values, however, unique to this study we have demonstrated that the levels of TGFb3 remained significantly lower in the TEA group through POD5 suggesting that overall the TEA group had lower systemic levels of immunosuppressive signaling in these patients.

The response to surgical insult also includes suppression of cell mediated immunity. The early postoperative period is characterized by a reduction in number and function of natural killer cells and cytotoxic T-cells [54], as well as a shift in Th1/Th2 signaling with suppression of Th1 cytokines [55]. Surrogate systemic markers for this process include IL12p70 [56–58] and CXCL10 [21]. Herein we found the expected suppression of CXCL10 in all patients and IL12p70 in the IV-PCA patients with preservation of IL12p70 systemic values on POD1 in TEA patients. The available literature suggests that compared to opioid based pain management, epidural analgesia does not maintain baseline IL12 levels [16, 18]. These studies, however, evaluated systemic IL12 levels including both the active heterodimer IL12p70 component involved in Th1 signaling and the less active IL12 subunits including IL12p40 and IL12p35. Hence we believe our specific analysis of IL12p70 is suggestive of preservation of Th1 signaling, and this is in keeping with data from other pre-clinical experiments [14] and randomized controlled trial demonstrating the preservation of cell mediated immunity with epidural analgesia [46, 47, 59, 60].

There are several limitations to this study. This is a subset analysis from a randomized controlled trial which included a smaller number of patients in the IV-PCA group compared to epidural group. This was in part due to the 2.5:1 randomization favoring epidural patients, but also limited by patients with available blood samples and further limited in the selection of patients with similar pathology and successful oncologic resection. This selection was prompted in order to have similar biomarker baselines values between patients with metastatic disease and comparable responses to inflammatory stimuli given the impact of tumor load on immune function [61]. The resulting subset analysis compared two groups with similar preoperative and postoperative factors associated with the inflammatory response, as demonstrated in Table 1, allowing for distinct evaluation of the effect of TEA vs. IV-PCA on the studied biomarkers in the postoperative period. Further analysis to consider the impact of alternative sources of inflammatory stimuli on comprehensive biomarker panels, such as the Charlson’s comorbidity index or postoperative infectious complications, should be considered, but are beyond the scope of this study [62]. In addition, previously published data from the original RCT (https://clinicaltrials.gov/ NCT01438476) demonstrated no differences in postoperative complications between patients allocated to the TEA or IV-PCA group.

Due to limited understanding of threshold values for pathologic significance of the studied biomarkers a sample size analysis based on meaningful change in these biomarkers could not be performed prior to this investigation [30]. The number of patients included in this analysis, however, is similar to other prospective trials investigating the impacts of regional analgesia on biomarker panels [17–19, 41]. Additional limitations include the poor signal quality noted in 11 of the 32 biomarkers. These assay deficits were unexpected given the well-established biomarker platform utilized in this study, particularly for biomarkers with known impacts from epidural analgesia in the perioperative setting such as VEGF [19] and IL10 [28]. Measurable time points may have been missed the temporal profile of some biomarkers given known early (<24 hours) postoperative peaks, and future biomarker studies should consider evaluation of biomarkers on the evening of surgery. Of note, the remaining traditional biomarkers (IL6, IL8, and MCP1) demonstrated reliable results with appropriate signal quality and predictable postoperative temporal variation based on literature review. Further, the comprehensive biomarker panel yielded novel impacts of epidural analgesia on biomarkers Pentraxin-3, IL12p70, and TGFb which support existing literature demonstrating that epidural analgesia may preserve postoperative oncological relevant immune function. Future studies to correlate these markers to other known markers of physiologic recovery from surgery are underway and will aid in further understanding of the mechanisms associated with regional analgesia.

## MATERIALS AND METHODS

### Patient selection

The patients in this study were part of the randomized controlled trial (RCT) conducted at University of Texas MD Anderson Cancer Center (https://clinicaltrials.gov/ NCT01438476) evaluating outcomes in patients undergoing liver resection with TEA vs. IV-PCA [63]. From the original trial database, patients were selected for this correlative study if they consented for additional blood draws and completed perioperative blood sample collection. In order to compare relative changes at each time point for individual perioperative biomarker levels, this subset analysis further selected patients with metastatic disease undergoing complete (R1 or R0) oncologic resection.

### Epidural and patient controlled analgesia regimens

Perioperative analgesia regimens for the TEA and IV-PCA groups have been previously described [63]. In brief, patients in the TEA group underwent preoperative epidural placement between the 5th and 10th thoracic intervertebral space and after a test dose, received epidural hydromorphone and lidocaine prior to incision, and then received continuous epidural infusion of bupivacaine and hydromorphone. This continued until the patient was tolerating a regular diet and could be transitioned to oral pain medications. Patients in the IV-PCA group underwent intra-operative and postoperative intravenous (IV) hydromorphone based analgesia. Additional non-opioid pain medications could be administered by the patient’s primary team or acute pain service.

### Clinicopathologic variables and postoperative recovery variables

Patient data including age, gender, American Society of Anesthesiologist physical status classification system (ASA) score, body mass index (BMI), receipt of preoperative chemotherapy within 90 days of surgery, portal vein embolization, extent of hepatectomy, surgical incision, operative time, estimated blood loss (EBL), perioperative transfusion, post-operative complication, histology of metastatic disease, opioid consumption (measured in morphine mg equivalents) and quality of pain control were collected. Major hepatectomy was defined by resection of 3 or more Couinaud segments [64]. Pain scores were recorded throughout the postoperative time period by numeric/visual pain scale (0–10), and inadequately controlled pain in the early post-operative period was defined as a pain score of 7 or greater reported in the first 48 hours.

### Biomarkers

Patients who agreed to blood draws underwent collection of venous blood at the preoperative visit (T0) within 2 weeks before surgery, as well as collection of venous blood on the mornings of postoperative day (POD) 1, POD 3, and POD5. Plasma samples were prepared and levels (pg/mL) were assessed using multiplex bead assay (Bio-Rad Laboratories, Hercules, CA, USA and EMD, Bioscience Research Reagents, Temecula, CA, USA) as previously described [65]. The 27 biomarkers (MIP-1b, IL-6, INFγ, IL-5, GM-CSF, TNFα, IL-2, IL-1b, IL-13, IL-4, MCP-1, IL-8, IL-10, G-CSF, VEGF, Resistin, IL-7, IL-12p70, IL-17, CXCL12, CXCL10, Omentin-1, Pentraxin-3, Galactin-3, TGFb1, TGFb2, and TGFb3) were selected by literature review for analysis given known variation with inflammatory stimuli and oncologic relevance in signaling pathways. The biomarkers sLeptin R, Vaspin, FGF-21, FGF-23, and PON-1 were included as portions of the multiplex bead assay. Acceptable signal quality to calculate the values (pg/mL) for these biomarkers was determined only by the values within the standards concentration range for each assay. To confirm reliability, all biomarker values were performed in duplicate on the established assay by a single research team member and all patient’s time point samples were arranged on to a single plate.

### Statistical analysis

Demographic, intraoperative, and postoperative data were analyzed and summarized using medians (interquartile ranges [IQRs]) or means (standard deviations or 95% confidence intervals) to account for outliers and non-normality in our description of study variables. Chi-squared test was used for categorical clinicopathologic variables, and Wilcoxon rank-sum tests were used for continuous clinicopathologic variables as well as to compare time point values of biomarkers between groups. Given that the oncological relevant value or “biologic effect size” of the studied biomarkers in the perioperative setting has not been established per systemic concentration or relative fold change to baseline with respect to the impact of analgesic modalities, the postoperative biomarker values were compared in traditional fashion according to systemic concentration [pg/mL] between groups at each time point as well as by considerations of individual patient fold change at each time point relative to preoperative baseline [66–68]. A *p* value < 0.05 was considered statistically significant. Data were analyzed using SPSS software (version 23).

## CONCLUSIONS

In this subset analysis of a randomized controlled trial comparing TEA vs. IV-PCA, circulating biomarker concentrations in patients undergoing TEA demonstrated a dampened early pro-inflammatory response to surgery as well as maintenance of markers of cell mediated immunity, while also demonstrating persistently lower levels of immunosuppressive signaling throughout the postoperative period in patients with epidural analgesia. These results add to a growing body of literature to support further investigation of systemic biomarkers with oncologic mechanisms and known variation with inflammatory stimuli to further understand how perioperative inflammation impacts oncologic outcomes. Novel to this study, Pentraxin3 demonstrated significant variation with both surgical stress and anesthetic technique and could be considered for future investigations aimed at evaluating techniques to dampen perioperative inflammation.
