# Correction: Impact of epidural analgesia on the systemic biomarker response after hepatic resection

**DOI:** 10.18632/oncotarget.27295

**Published:** 2019-10-29

**Authors:** Diego Vicente, Miguel Patino, Rebecca Marcus, Heather Lillemoe, Preparim Limani, Timothy Newhook, Andy Lee, Ching-Wei Tzeng, Yun Segraves-Chun, David Tweardy, Vijaya Gottumukkala, Jean-Nicolas Vauthey, Thomas Aloia, Juan P. Cata

**Affiliations:** ^1^ Department of Surgical Oncology, The University of Texas MD Anderson Cancer Center, Houston, TX, USA; ^2^ Anesthesiology and Surgical Oncology Research Group, Houston, TX, USA; ^3^ Department of Molecular and Cellular Oncology, The University of Texas MD Anderson Cancer Center, Houston, TX, USA; ^4^ Department of Anesthesiology and Perioperative Medicine, The University of Texas MD Anderson Cancer Center, Houston, TX, USA

**This article has been corrected:** The last name of the 4th author was spelled incorrectly. The proper spelling is given below:

**Heather Lillemoe^1^**

Original article: Oncotarget. 2019; 10:584–594. 584-594. https://doi.org/10.18632/oncotarget.26549

